# Pollen-mediated gene flow from transgenic cotton is constrained by physical isolation measures

**DOI:** 10.1038/s41598-018-21312-1

**Published:** 2018-02-12

**Authors:** Shuo Yan, Weilong Zhu, Boyu Zhang, Xinmi Zhang, Jialin Zhu, Jizhe Shi, Pengxiang Wu, Fengming Wu, Xiangrui Li, Qingwen Zhang, Xiaoxia Liu

**Affiliations:** 1 Department of Entomology, China Agricultural University, Beijing, 100193 P.R. China; 2 National Agricultural Technology Extension and Service Center, Beijing, 100125 P.R. China; 3 Liuzhou Agriculture Technology Extend Service Center, Liuzhou, 545002 P.R. China; 4 Department of Entomology and Plant Pathology, Auburn University, Auburn, Alabama 36830 USA; 5 Beijing Entry-Exit Inspection and Quarantine Bureau, Beijing, 100026 P.R. China; 6 Department of Entomology, University of Kentucky, Lexington, KY 40546 USA; 7 Institute of Zoology, Chinese Academy of Sciences, Beijing, 100101 P.R. China; 8 Institute of Plant Protection, Chinese Academy of Agricultural Sciences, Beijing, 100193 China

## Abstract

The public concern about pollen-mediated gene flow (PGF) from genetically modified (GM) crops to non-GM crops heats up in recent years over China. In the current study, we conducted greenhouse and field experiments to measure PGF with various physical isolation measures, including 90, 80, 60 and 40 holes/cm^2^ separation nets and *Sorghum bicolor*, *Zea mays* and *Lycopersicon esculentum* separation crops between GM cotton and non-GM line (Shiyuan321) by seed DNA test during 2013 to 2015, and pollen grain dyeing was also conducted to assess the pollen flow in greenhouse during 2013. Our results revealed that (1) PGF varied depending on the physical isolation measures. PGF was the lowest with 90 holes/cm^2^ separation net and *S*. *bicolor* separation crop, and the highest with 40 holes/cm^2^ separation net and no isolation measure. (2) Similar to PGF results, 90 holes/cm^2^ separation net and *S*. *bicolor* separation crop could minimize the pollen dispersal. (3) PGF declined exponentially with increasing distance between GM cotton and Shiyuan321. Because of the production mode of farm household (limited cultivated area) in China, our study is particularly important, which is not only benefit for constraining PGF, but also has potential application value in practical production and the scientific researches.

## Introduction

Genetic engineering has been used for the introduction of new traits to specific crops^1–5^, which is a good method to breed new and improved cultivars, and satisfies the increasing consumer demand for a consistent supply of high-quality crops with fewer blemishes from insects and diseases and reduced pesticide residues^6,7^. The steady growth of genetically modified (GM) plants in China attests to their advantages for producers^8–11^, and “No. 1 document” of central government in 2016 first mentioned that GM technology should be cautious introduced in agriculture, showing the determination to extend GM crops in China. However, public concerns have been raised about the environmental and food safety of GM crop planting^3,12–14^. The “No. 1 document” in 2017 did not mention the GM technology after the positive policies of past 3 years, revealing the big hurdle in applying the GM technology over the China.

One of the concerns to introduce GM crops into environment is the flow of transgene-containing pollen to the surroundings^15–17^. Gene flow itself is neutral and usually occurs between conventional crops. Gene flow is of great importance in seed production in conventional crops, and the effect of gene flow depends on the differences between the recipient and donor plants^3,6,11^. Pollen-mediated gene flow (PGF) makes the movement of introduced genes from GM crops to other plants possible, which usually occurs if GM pollen is delivered to the receptive stigma by insects or wind^12,18–21^. Thus, limiting the dispersal of GM pollen is the key to prevent cross-pollination between GM and non-GM crops^5,22,23^. Physical isolation measure (separation net and crop) is an effective way to constrain pollen flow with low activity of pollinators, which in turn constrains the PGF^24–27^. Meanwhile, physical isolation distance is also important for attenuating the gene flow risk because of the decline of PGF with increasing distance^14,21,26,28,29^. What has been less studied is the effectiveness of various isolation measures to constrain PGF. Unlike the developed countries, farmers in China possess relatively small cultivated area, even less than 500 m^2^ for per farmer, and the phenomenon that farmers select different crop varieties is very common. Thus, the coexistence of GM and non-GM crops is very likely in China, which increases the risk of PGF. Our study determining the effectiveness of various isolation measures to constrain PGF is particularly important in China, which is not only benefit for decreasing the risk of PGF, but also has potential application value in practical production and the scientific researches of GM crops.

Cotton (*Gossypium hirsutum* L.) is primarily grown as an irrigated annual crop in the Changjiang River Region, the Yellow River Region and the North-western Region of China. GM cotton, producing insecticidal proteins from *Bacillus thuringiensis* (Bt) was first introduced in China in 1997 to control lepidopteran pests^30,31^. Previous studies have shown that PGF in cotton could be greatly limited by low activity of pollinators, isolation distance and low speed of wind^19,21,32,33^. Long isolation distance cannot be applied in practical production in China because of the limited cultivated area. In the present study, we measured pollen dispersal distance and PGF in GM cotton with 90, 80, 60 and 40 holes/cm^2^ separation nets and *Sorghum bicolor*, *Zea mays* and *Lycopersicon esculentum* separation crops by pollen grain dyeing method and molecular techniques respectively to determine the effectiveness of various physical isolation measures to constrain pollen movement and PGF. Our goal was to find an effective physical isolation measure to constrain PGF.

## Results

### Greenhouse and field observation

The agronomic behavior of GM cotton and Shiyuan321 was similar between the greenhouse and field trials, and flowering was synchronous between two types of cottons throughout the flowering period, thus ensuring ample opportunity for pollen transfer. In greenhouses, wind velocities (most distance points: 0.5–0.6 m/s) were stable (Figure S1). In field, no pesticide was used to control pests, and *Apolygus lucorum*, *Aphis gossypii*, *Apis mellifera*, *Pieris rapae* and *Harmonia axyridis* were observed in both years.

### Evaluation of pollen dispersal with different physical isolation measures

Pollen grain dyeing was conducted in a greenhouse during 2013 to assess the pollen flow with various physical isolation measures. As shown in Table 1, dyed pollens with various separation nets and crops were observed most at the distance of 3.2 and 4.0 m, respectively. The 90 holes/cm^2^ separation net and *S*. *bicolor* separation crop could minimize the pollen dispersal, whereas the constraints of pollen dispersal by 40 holes/cm^2^ separation net and *L*. *esculentum* separation crop were the weakest, and dyed pollens were observed at all distance points with no isolation measure (CK).

**Table 1:** The comparison of dyed pollen number with different physical isolation measures in greenhouse during 2013.

Distance (m)	90 holes/cm^2^	80 holes/cm^2^	60 holes/cm^2^	40 holes/cm^2^
0.8	1	1	2	2
1.6	2	2	2	7
3.2	5	10	11	12
6.4	1	0	4	7
12.8	0	0	2	4
19.2	0	0	3	2
25.6	0	0	2	2
Total	9	13	26	36
	***S***. ***bicolor***	***Z***. ***mays***	***L***. ***esculentum***	**CK**
4	4	5	10	15
4.8	1	1	5	9
6.4	1	3	4	4
9.6	0	2	2	7
16.0	0	0	1	3
22.4	0	0	1	2
28.8	0	0	0	3
40.0	0	0	0	1
Total	6	11	23	44

### Assessment of pollen-mediated gene flow in greenhouse experiment

A total of 7,200 seeds (60 seeds/sample × 4 separation nets × 7 distances × 2 yr + 60 seeds/sample × 4 separation crops × 8 distances × 2 yr) were sampled to assess PGF by DNA analyses in greenhouse experiment.

**Figure 1:**
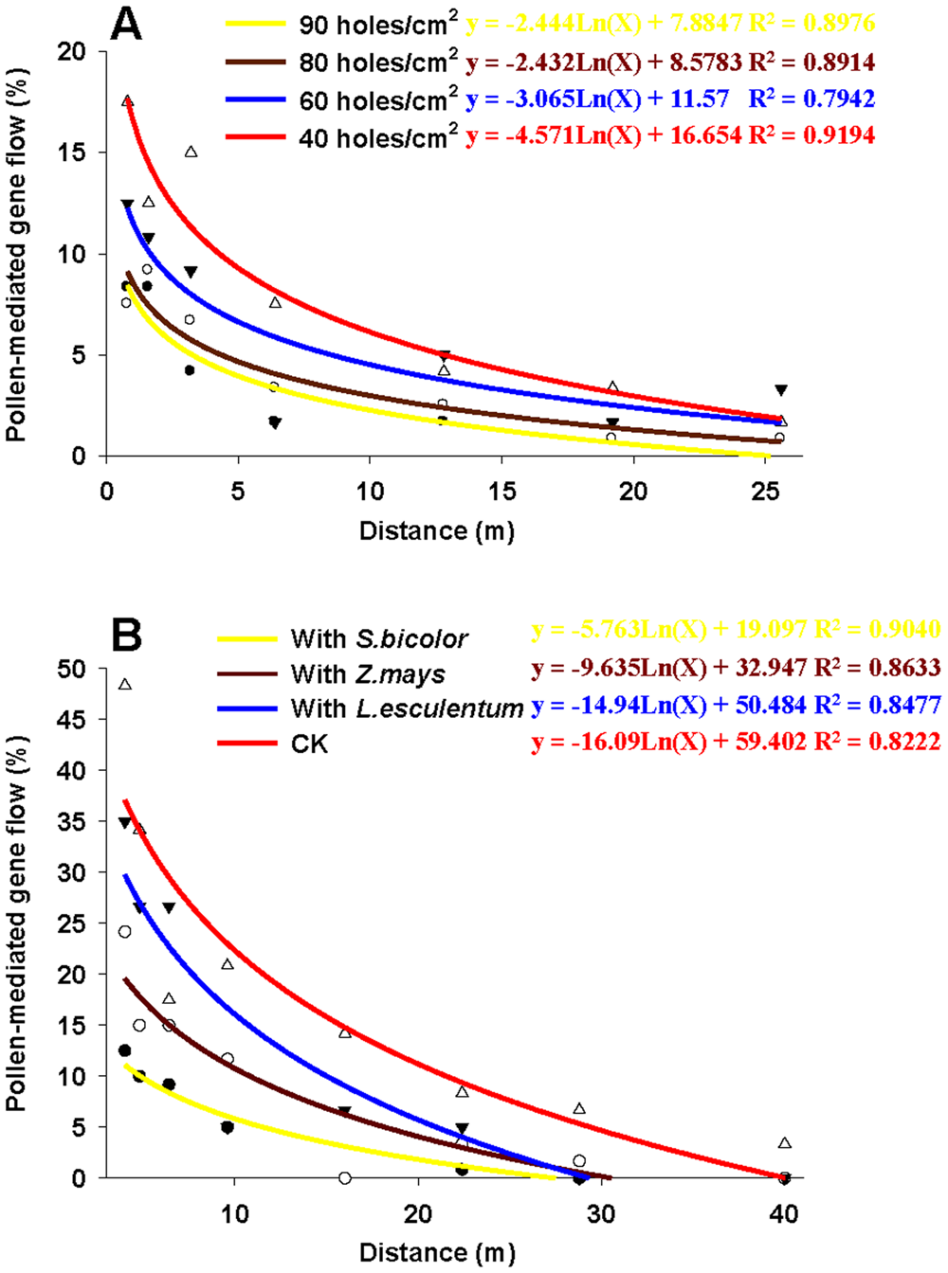
Regression analysis for PGF with different separation nets (**A**) and crops (**B**) in greenhouse experiment as a function of the distance. The determination coefficient (R^2^) between PGF and distance was obtained using SigmaPlot 10.0 software. Regression analysis was based on the data of the 2 yr study.

Hybridization was detected during the 2 yr of the study, and varied by the physical isolation measure and distance (Table 2). More specifically, the average PGF of all sampled distances during the 2 yr were 3.69%, 4.40%, 6.31% and 8.81% with 90, 80, 60 and 40 holes/cm^2^ separation nets, respectively (*F*_3,164_ = 4.534, *P* = 0.004), 4.69%, 8.85%, 13.13% and 19.17% with *S*. *bicolor*, *Z*. *mays*, *L*. *esculentum* separation crops and no isolation measure (CK), respectively (*F*_3,188_ = 10.766, *P* < 0.001). Net isolation (5.80%) was more effective to constrain PGF than crop isolation (8.89%) (*t* = 2.710, df = 226.845, *P* = 0.007). As shown in Table 2 and Fig. 1, PGF tended to decline with increasing distance, ranging from 16.67% at 0.8 and 3.2 m to 0.00% at 19.2 m in 2013 and from 18.33% at 0.8 m to 0.00% at 25.6 m in 2014 with 40 holes/cm^2^ separation net, from 43.33% at 4.0 m to 6.67% at 28.8 and 40.0 m in 2013 and from 53.33% at 4.0 m to 0.00% at 40.0 m in 2014 with no isolation measure (CK). Line (y = lnx) was the best-fit regression curve in Fig. 1 (90 holes/cm^2^ R^2^ = 0.8976; 80 holes/cm^2^ R^2^ = 0.8914; 60 holes/cm^2^ R^2^ = 0.7942; 40 holes/cm^2^ R^2^ = 0.9194; *S*. *bicolor* R^2^ = 0.9040; *Z*. *mays* R^2^ = 0.8633; *L*. *esculentum* R^2^ = 0.8477; CK R^2^ = 0.8222).

**Table 2:** Pollen-mediated gene flow frequency with different physical isolation measures in greenhouse during 2013 and 2014 (%).

Distance (m)	90 holes/cm^2^	80 holes/cm^2^	60 holes/cm^2^	40 holes/cm^2^
**2013**				
0.8	10.00 ± 5.77	6.67 ± 3.33	11.67 ± 4.41	16.67 ± 4.41
1.6	6.67 ± 4.41	8.33 ± 4.41	13.33 ± 4.41	15.00 ± 5.00
3.2	3.33 ± 1.67	8.33 ± 3.33	8.33 ± 3.33	16.67 ± 4.41
6.4	3.33 ± 3.33	3.33 ± 1.67	3.33 ± 1.67	8.33 ± 1.67
12.8	0.00 ± 0.00	1.67 ± 1.67	3.33 ± 3.33	3.33 ± 1.67
19.2	0.00 ± 0.00	0.00 ± 0.00	0.00 ± 0.00	0.00 ± 0.00
25.6	1.67 ± 1.67	1.67 ± 1.67	6.67 ± 1.67	3.33 ± 3.33
Average	3.57 ± 1.25	4.29 ± 1.11	6.67 ± 1.39	9.05 ± 1.81
	***S***. ***bicolor***	***Z***. ***mays***	***L***. ***esculentum***	**CK**
4	6.67 ± 3.33	20.00 ± 5.77	33.33 ± 6.67	43.33 ± 9.28
4.8	13.33 ± 6.01	13.33 ± 6.01	21.67 ± 7.27	35.00 ± 7.64
6.4	8.33 ± 4.41	6.67 ± 6.67	26.67 ± 6.67	15.00 ± 5.00
9.6	5.00 ± 2.89	16.67 ± 4.41	3.33 ± 3.33	16.67 ± 3.33
16.0	0.00 ± 0.00	0.00 ± 0.00	3.33 ± 3.33	18.33 ± 4.41
22.4	1.67 ± 1.67	6.67 ± 3.33	6.67 ± 3.33	10.00 ± 0.00
28.8	0.00 ± 0.00	0.00 ± 0.00	0.00 ± 0.00	6.67 ± 3.33
40.0	0.00 ± 0.00	0.00 ± 0.00	0.00 ± 0.00	6.67 ± 4.41
Average	4.38 ± 1.32	7.92 ± 1.99	11.88 ± 2.93	18.96 ± 3.05
**2014**				
0.8	6.67 ± 6.67	8.33 ± 4.41	13.33 ± 3.33	18.33 ± 6.01
1.6	10.00 ± 5.77	10.00 ± 5.77	8.33 ± 4.41	10.00 ± 5.77
3.2	5.00 ± 2.89	5.00 ± 5.00	10.00 ± 5.77	13.33 ± 6.67
6.4	0.00 ± 0.00	3.33 ± 3.33	0.00 ± 0.00	6.67 ± 6.67
12.8	3.33 ± 3.33	3.33 ± 3.33	6.67 ± 6.67	5.00 ± 2.89
19.2	1.67 ± 1.67	1.67 ± 1.67	3.33 ± 3.33	6.67 ± 3.33
25.6	0.00 ± 0.00	0.00 ± 0.00	0.00 ± 0.00	0.00 ± 0.00
Average	3.81 ± 1.42	4.52 ± 1.42	5.95 ± 1.68	8.57 ± 2.02
	***S***. ***bicolor***	***Z***. ***mays***	***L***. ***esculentum***	**CK**
4	18.33 ± 4.41	28.33 ± 6.01	36.67 ± 6.01	53.33 ± 9.28
4.8	6.67 ± 3.33	16.67 ± 8.82	31.67 ± 1.67	33.33 ± 7.26
6.4	10.00 ± 5.77	23.33 ± 3.33	26.67 ± 6.01	20.00 ± 0.00
9.6	5.00 ± 2.89	6.67 ± 3.33	6.67 ± 6.67	25.00 ± 7.64
16.0	0.00 ± 0.00	0.00 ± 0.00	10.00 ± 5.77	10.00 ± 5.77
22.4	0.00 ± 0.00	0.00 ± 0.00	3.33 ± 3.33	6.67 ± 4.41
28.8	0.00 ± 0.00	3.33 ± 3.33	0.00 ± 0.00	6.67 ± 6.67
40.0	0.00 ± 0.00	0.00 ± 0.00	0.00 ± 0.00	0.00 ± 0.00
Average	5.00 ± 1.56	9.79 ± 2.56	14.38 ± 3.20	19.38 ± 3.87

Means ± SE. Seeds from lower, middle, and upper flowers were set up as 3 replications.

### Assessment of pollen-mediated gene flow in field experiment

A total of 5,040 seeds (60 seeds/sample × 3 physical isolation measures × 7 distances × 2 directions × 2 yr) were sampled to assess PGF by DNA analyses in field experiment.

**Figure 2:**
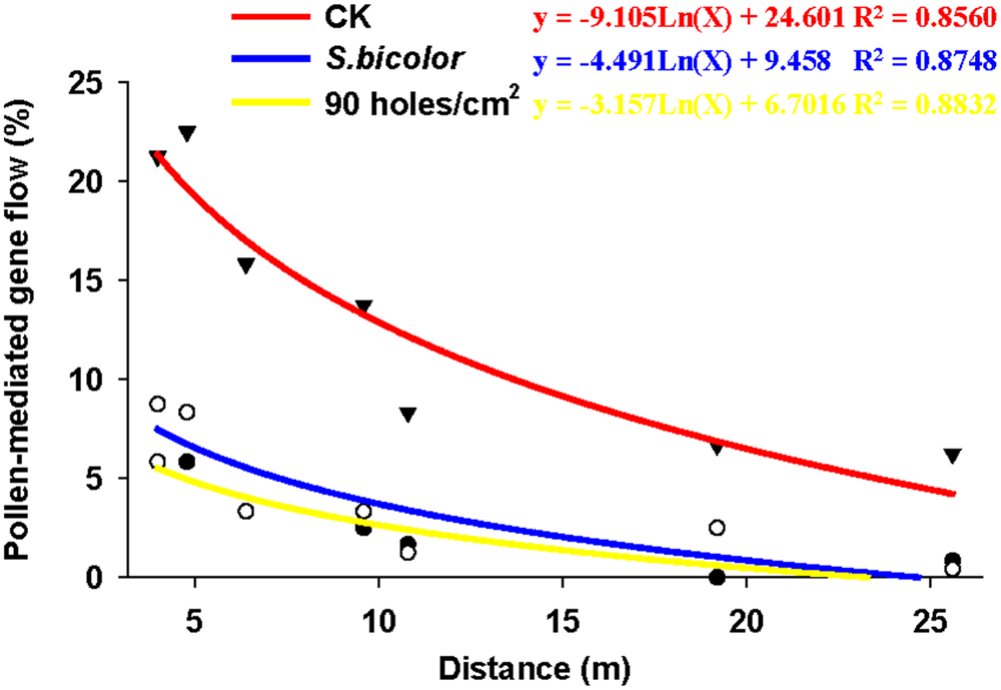
Regression analysis for PGF with different physical isolation measures in field experiment as a function of the distance. The determination coefficient (R^2^) between PGF and distance was obtained using SigmaPlot 10.0 software. Regression analysis was based on the data of the 2 yr study.

Similar to greenhouse experiment, hybridization was varied by the physical isolation measure and distance in field experiment. More specifically, the average PGF of all sampled distances during the 2 yr were 2.86%, 3.99% and 13.51% with 90 holes/cm^2^ separation net, *S*. *bicolor* separation crop and no isolation measure (CK), respectively (*F*_2,249_ = 51.017, *P* < 0.001), however PGF with net isolation and crop isolation shown no significant difference (*t* = 1.342, df = 166, *P* = 0.182)(Table 3). As shown in Table 3 and Fig. 2, PGF also declined with increasing distance, ranging from 29.17% at 4.8 m to 6.67% at 25.6 m in 2014 and from 22.50% at 4.0 m to 5.83% at 19.2 and 25.6 m in 2015 with no isolation measure (CK), whereas PGF with 90 holes/cm^2^ separation net and *S*. *bicolor* separation crop were relatively low among different distance points. Line (y = lnx) was the best-fit regression curve in Fig. 2 (CK R^2^ = 0.8560; *S*. *bicolor* R^2^ = 0.8748; 90 holes/cm^2^ R^2^ = 0.8832).

**Table 3:** Pollen-mediated gene flow frequency with different physical isolation measures in field during 2014 and 2015 (%).

Distance (m)	90 holes/cm^2^	*S*. *bicolor*	CK
**2014**			
4	6.67 ± 2.47	5.00 ± 2.58	20.00 ± 2.89
4.8	3.33 ± 2.11	8.33 ± 3.33	29.17 ± 3.96
6.4	5.00 ± 2.58	3.33 ± 2.11	15.83 ± 3.75
9.6	1.67 ± 1.05	5.00 ± 2.58	10.83 ± 3.00
10.8	1.67 ± 1.67	1.67 ± 1.05	9.17 ± 3.75
19.2	0.00 ± 0.00	3.33 ± 1.67	7.50 ± 2.81
25.6	0.00 ± 0.00	0.00 ± 0.00	6.67 ± 3.33
Average	2.62 ± 0.71	3.81 ± 0.85	14.17 ± 1.67
**2015**			
4	5.00 ± 2.58	12.50 ± 3.82	22.50 ± 4.23
4.8	8.33 ± 3.33	8.33 ± 3.33	15.83 ± 4.17
6.4	1.67 ± 1.05	3.33 ± 2.11	15.83 ± 4.17
9.6	3.33 ± 1.67	1.67 ± 1.05	16.67 ± 3.33
10.8	1.67 ± 1.05	0.83 ± 0.83	7.50 ± 3.59
19.2	0.00 ± 0.00	1.67 ± 1.67	5.83 ± 2.39
25.6	1.67 ± 1.67	0.83 ± 0.83	5.83 ± 3.00
Average	3.03 ± 0.78	4.17 ± 1.02	12.86 ± 1.57

Means ± SE. Seeds from lower, middle, upper flowers in southern and northern fields were set up as 6 replications.

## Discussion

Efficient pollen dispersal leading to the fertilization, is the major problem when considering the risk of transgene escape in particular for GM crops that can hybridize with non-GM lines or related wild species^1,34^. When the desired gene gets introgressed with other species by recombination, particularly weedy relatives and feral and volunteer crop plants, there is a potential risk for new and more invasive weeds to arise^35–37^, and domestication traits can also make weeds less invasive^38^. Regulation approaches of GM crops in Europe are mainly based on the process of making GM crops, whereas those of Canada are based on the GM product. The regulation of GM crops in USA focuses primarily on the characteristics of the product, and a useful oversight of the regulatory process is maintained. China implements a relative pragmatic approach to GM crop regulations, which are basically based on the products and depended on the economic interest of a given application^3^. Thus, preventing gene flow is important for risk management of releasing GM crops, and the physical isolation measure may be a good choice in practical production to constrain the pollen dispersal^24–27^. In the present study, we determined the pollen dispersal with various physical isolation measures and measured PGF between the GM cotton and non-GM cotton in greenhouse and field experiments during two years using the molecular technique, which was confirmed to be more reliable to determine PGF than biological assays because of the weaker resistance of hybrids for some assays^21^. Previous studies on PGF were mainly conducted in fields^14,39–41^, whereas our study was conducted in greenhouse and field, which provided a more complementary assessment.

Zhang *et al*.^8^ found that PGF from GM cotton decreased exponentially as dispersal distance increased, ranging from 10.13% at 1 m to 0.04% at 50 m for the *tfd* A gene, 8.16% at 1 m to 0.08% at 20 m for the *Bt* gene. Our early study^21^ found that PGF with Shiyuan321 went from 30% to <5% within 1.6 m from the test plot, and a low level from 8.33% to <1% PGF continued to occur sporadically out to a distance of 25.6 m. Van Deynze *et al*.^33^ found PGF was independent of direction from the source plot and declined exponentially with increasing distance from 7.65% at 0.3 m to <1% beyond 9 m with *A*. *melifera*. In the present study, we collected a total of 12,240 cotton seeds during the 2-yr study and then extracted DNA from seeds directly to determine PGF. Similar to other previous studies^14,26,42^, we found PGF with any isolation measure in greenhouse and field tended to decline significantly with increasing distance because of the decreased proportion of GM pollens over the distance.

The heights of *S*. *bicolor*, *Z*. *mays* and *L*. *esculentum* were nearly 3.2, 2.2 and 1.5 m respectively at the pollination of cottons. Cotton pollen diameter is 120–150 μm, and the hole sizes of four kind separation nets were bigger than the size of cotton pollen. Our results revealed that 90 holes/cm^2^ separation net and *S*. *bicolor* separation crop could constrain the pollen dispersal effectively, whereas dyed pollens were observed at all distance points with no isolation measure (CK). The constraint of pollen dispersal was benefit for reducing the environment risk of transgene escape. Similar to pollen grain dyeing results, we found that PGF in greenhouse and field was constrained by various physical isolation measures, with 90 holes/cm^2^ separation net and *S*. *bicolor* separation crop having lower PGF rates than other isolation measures. Meanwhile net isolation was more effective to constrain PGF than crop isolation no matter in greenhouse and field. The height of separation crop might be the key to constrain PGF. In general, the 20 m cotton buffer or 50 m bare ground is accepted to provide sufficient containment of GM cotton in small scale trials in Australia, compared to sometimes up to 800 m for foundation seed production with additional pollen buffer rows surrounding the crop in USA^18^. Zhang *et al*.^8^ confirmed that the farthest distance of pollen dispersal from cotton was 50 m, and recommended a 60 m buffer zone to limit pollen dispersal from small-scale field tests. However, isolation distance requirement may vary under different environmental conditions, especially wind and pollinator activity^14,19,21,33^. In the present study, we could still detect the gene flow occurring at 25.6 m both with 90 holes/cm^2^ separation net and *S*. *bicolor* separation crop in field, and the further isolation distance with *S*. *bicolor* buffer or double separation nets may be needed to constrain PGF in agricultural production and scientific researches. The application of nets may be also benefit for controlling many insect plagues, whereas the disadvantage of this method is the high expense, and the price of nets increases with the number of holes/cm^2^. The application of long isolation distance to constrain PGF is unrealistic in China because of the limited cultivated area, and agricultural ministries of each level should explore subsidy mechanics to promote the application of plant buffer or other physical isolation measures in practical production.

We observed that PGF occurred at a higher frequency in greenhouse than field during our 2-yr study, which might be related to the automatic wind in greenhouse. PGF or cross-fertilization in field might be more complex than those in greenhouse, which was related with changeable natural condition^19,21,33,43,44^. Meanwhile, the planting scale of GM crops and the genetic relationship between GM crops and adjacent crops would also influence PGF^21,45^. Thus, we should consider the local natural condition and agroecological environment before setting the proper isolation measure to constrain PGF. The development of transgenic technology in China has already drawn heated public concerns about the possible risk of GM food in recent years, and central government has a long way to extend consuming transgenic crops. The physical isolation measure will play a more important role in future agricultural production.

## Methods

### Plant materials

The inbred line Shiyuan321 (pollen receptor) and GM cotton (pollen donor) were obtained from the Cotton Research Institute, Chinese Academy of Agricultural Sciences, and were purified by consecutive two-generations of self-crosses, to ensure that each line was homozygous. The GM cotton was obtained by transferring the insect resistance gene *Cry I Ac* and the glyphosate resistance gene *CP4 EPSPS* from *Agrobacterium tumefaciens* into the embryonic calli of Shiyuan321 via microprojectile bombardment. The hemizygous progeny can be easily identified by PCR analyses because of the high resistance gene expression^23,46–48^.

### Greenhouse layout and procedures

To evaluate the impacts of physical isolation measures on PGF, the greenhouse trials (L60 m × W8 m × H4 m) were carried out in the Shangzhuang Experimental Station of China Agricultural University (40°14′N, 116°19′E), Beijing, China during 2013 and 2014. Greenhouse provided a relatively closed environment, which was not benefit for pollen dispersal. Thus, several electric fans (2 m height) were installed every 4.5 m, turned on (from 6:00 to 18:00) to promote pollen dispersal, and wind speeds were monitored at 8:00, 12:00 and 16:00 (as 3 replications) using an anemograph (ZRQF-F303, Beijing Detector Instrument Co., Beijing, China)^19^. The agronomic behavior of GM cotton and Shiyuan321 was similar to a standard cultivar in the field. Plants were monitored at least twice weekly for florescence emergence and anthesis from early July through late August (24–29 °C, full-bloom stage for all plants) to ensure the flowering synchrony between two types of cottons^49^. Treatment rows were spaced 0.8 m apart and plants within rows were spaced 0.5 m apart.

### Separation net treatment

**Figure 3:**
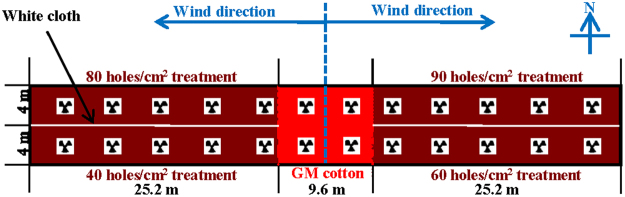
Greenhouse trial design to measure the impacts of different separation nets on pollen-mediated gene flow during 2013 and 2014. The GM cotton was planted in a 9.6 m × 8 m square in the middle of a 60 m × 8 m square field of Shiyuan321. Electric fans were installed to promote pollen dispersal.

As shown in Fig. 3, 90, 80, 60 and 40 holes/cm^2^ separation nets (3 m height) were installed in one greenhouse to evaluate the isolation effect. Different treatments were separated by white cloth from the ceiling to the ground. The GM cotton was planted in a 9.6 m × 8 m square in the middle of a 60 m × 8 m square area of Shiyuan321. Pollen grain dyeing was conducted during the full-bloom stage to measure the pollen dispersal according to the method of Wang *et al*.^50^ during 2013. Pollens from 15 upper flowers of the first row GM cottons were dyed with erythrosine, and dyed pollens on flowers were counted at the distance of 0.8, 1.6, 3.2, 6.4, 12.8, 19.2 and 25.6 m from GM cottons after 3 days. No significant differences in PGF were observed for flower positions on the pollen receptors^32^, so seeds from pollen receptors were obtained at harvest by hand from lower, middle, and upper flowers as 3 replications at the distance of 0.8, 1.6, 3.2, 6.4, 12.8, 19.2 and 25.6 m for PGF evaluation when flower mature but before excessive shattering^33^.

### Separation crop treatment

**Figure 4:**
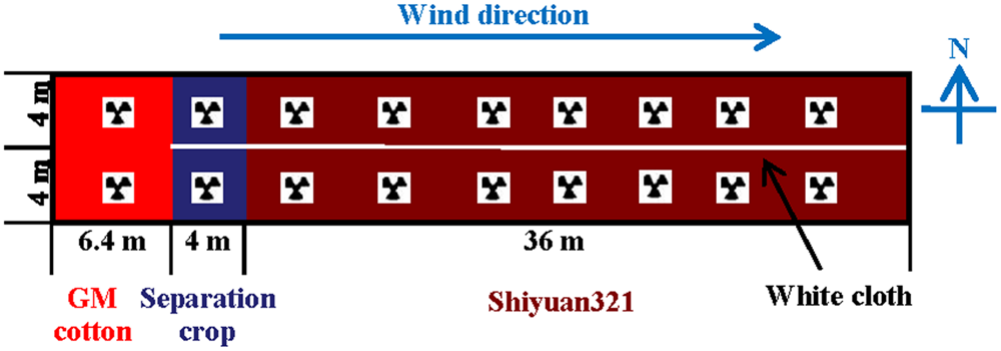
Greenhouse trial design to measure the impacts of different separation crops on pollen-mediated gene flow during 2013 and 2014. The separation crop was planted in a 4 m × 8 m square in the middle of a 46.4 m × 8 m square field of cotton. Electric fans were installed to promote pollen dispersal.

As shown in Fig. 4, *Sorghum bicolor* (Yajin1), *Zea mays* (Nongda80) and *Lycopersicon esculentum* (Zhefen702) separation crops and no isolation measure (CK) were set up in two greenhouses to evaluate the isolation effect. Different treatments were separated by white cloth from the ceiling to the ground. Treatment rows of separation crops were spaced 0.8 m apart and plants within rows were spaced 0.5 m apart. The separation crop was planted in a 4 m × 8 m square in the middle of a 46.4 m × 8 m square area of cotton. Pollen grain dyeing was similar to separation net treatment, and dyed pollens were counted at the distance of 4, 4.8, 6.4, 9.6, 16.0, 22.4, 28.8 and 40.0 m. At harvest, seeds from pollen receptors were obtained at the distance of 4, 4.8, 6.4, 9.6, 16.0, 22.4, 28.8 and 40.0 m for PGF evaluation.

### Design and maintenance of experimental field plot

**Figure 5:**
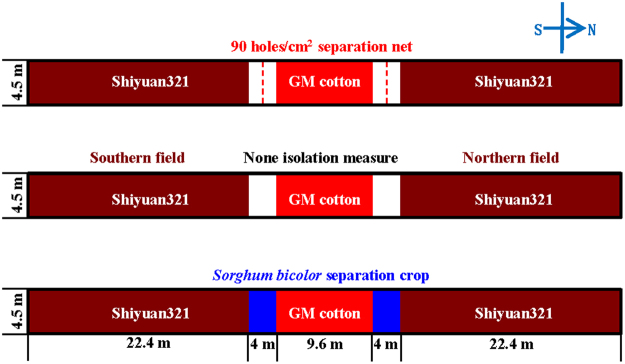
Field trial design to measure the impacts of different physical isolation measures on pollen-mediated gene flow during 2014 and 2015. The 90 holes/cm^2^ separation net, no isolation measure (CK) and *S*. *bicolor* separation crop were set up to evaluate the isolation effect. The GM cotton was planted in a 9.6 m × 4.5 m square in the middle of a 62.4 m × 4.5 m square field.

The field trial was also carried out in the Shangzhuang Experimental Station during 2014 and 2015. Treatment rows of both cottons and separation crops were spaced 0.8 m apart and plants within rows were spaced 0.5 m apart. As shown in Fig. 5, the 90 holes/cm^2^ separation net (3 m height), no isolation measure (CK) and *S*. *bicolor* separation crop were set up to evaluate the isolation effect. The GM cotton was planted in a 9.6 m × 4.5 m square in the middle of a 62.4 m × 4.5 m field. At harvest, seeds from pollen receptors were obtained from lower, middle, and upper flowers as 3 replications at the distance of 4, 4.8, 6.4, 9.6, 12.8, 19.2 and 25.6 m for PGF evaluation.

### PGF evaluation

For each distance point, 60 seeds [20 seeds from each height of flowers (lower, middle, and upper)] were screened for hybrid confirmation by polymerase chain reaction (PCR) amplification of the fragment sequence of *Cry I Ac*. DNA was extracted using the cetyltrimethyl ammonium bromide (CTAB) protocol^51^ with the addition of 20 g/L polyvinylpyrrolidone (PVP) in the extraction buffer. A 1 μL sample of DNA was used to perform spectroscopic quantitation using a NanoDrop 2000 spectrophotometer (Thermo Fisher, USA). Then, 80 ng DNA samples were used to amplify the target gene with primers of 5′-GAAGGATTGAGCAATCTCTAC-3′ and 5′-CAATCAGCCTAGTAAGGTCGT-3′^19^. The PCR reaction was typically cycled 30 times at 94 °C for 60 s, 56 °C for 60 s, and 72 °C for 90 s^19^. Routine screening for PCR assay was performed by agarose gel electrophoresis (10 g agarose/L) with GM cotton as positive control and Shiyuan321 as negative control.

$${\rm{Gene}}\,{\rm{flow}}\,( \% )=[({\rm{number}}\,{\rm{of}}\,{\rm{seed}}\,{\rm{progeny}}\,{\rm{expressing}}\,{\rm{Cry}}\,{\rm{I}}\,{\rm{Ac}})/({\rm{number}}\,{\rm{of}}\,{\rm{tested}}\,{\rm{seeds}})]\times 100$$

### Statistical analysis

All statistical analyses were conducted using the SPSS 16.0 software (IBM, Armonk, NY). All descriptive statistics were given as the mean values and standard errors of the mean (mean ± SE). The data were analyzed using Tukey HSD test and intendent *t* test at the *P* = 0.05 level of significance. Regression analyses (a potential dispersal model: y = lnx) were performed with SigmaPlot 10.0 software (Systat Software Inc., San Jose), and the determination coefficient (R^2^) between PGF and distance was obtained from SigmaPlot 10.0 software.

## Electronic supplementary material

Supplementary information
