# Supplementary material for: Pollen-mediated gene flow from transgenic cotton is constrained by physical isolation measures
Source: Sci Rep. 2018 Feb 12;8:2862. doi: 10.1038/s41598-018-21312-1 (PMC5809611; doi:10.1038/s41598-018-21312-1)

Pollen-mediated gene flow from transgenic cotton is constrained by physical isolation measures

Shuo Yan[1,2], Weilong Zhu[3], Boyu Zhang[1], Xinmi Zhang[1,4], Jialin Zhu[5], Jizhe Shi[1,6], Pengxiang Wu[1,7], Fengming Wu[1,7], Xiangrui Li[8], Qingwen Zhang[1] and Xiaoxia Liu[1*]

[1]Department of Entomology, China Agricultural University, Beijing, 100193, P.R. China

[2]National Agricultural Technology Extension and Service Center, Beijing, 100125, P.R. China

[3]Liuzhou Agriculture Technology Extend Service Center, Liuzhou, 545002, P.R. China

[4]Department of Entomology and Plant Pathology, Auburn University, Auburn, Alabama, 36830, USA

[5]Beijing Entry-Exit Inspection and Quarantine Bureau, Beijing, 100026, P.R. China

[6]Department of Entomology, University of Kentucky, Lexington, KY, 40546, USA

[7]Institute of Zoology, Chinese Academy of Sciences, Beijing, 100101, P.R. China

[8]Institute of Plant Protection, Chinese Academy of Agricultural Sciences, Beijing, 100193, China

*Correspondence and requests for materials should be addressed to X. L. (email: liuxiaoxia611@cau.edu.cn)

Supplementary legends

Figure S1. The measurement of wind velocity in various treatments.

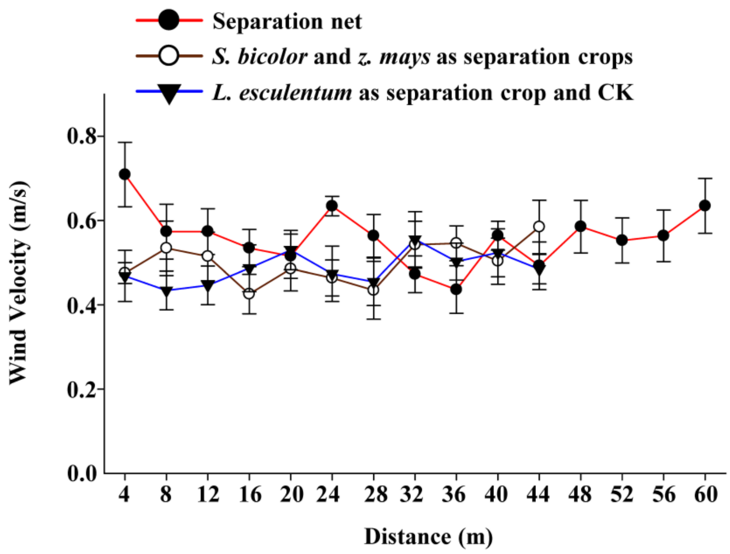

Supplement: Supplementary file 1 — Supplementary information [file 41598_2018_21312_MOESM1_ESM.pdf]
